# Reversible ubiquitylation in plant biology

**DOI:** 10.3389/fpls.2014.00707

**Published:** 2014-12-10

**Authors:** Hongyong Fu, Daphne R. Goring, Pascal Genschik

**Affiliations:** ^1^Institute of Plant and Microbial Biology, Academia SinicaTaipei, Taiwan; ^2^Cell and Systems Biology, University of TorontoToronto, ON, Canada; ^3^Institut de Biologie Moléculaire des Plantes, Centre National de la Recherche ScientifiqueStrasbourg, France

**Keywords:** ubiquitin, histone, self-incompatibility, deubiquitination, ubiquitin ligase, abiotic stress, plant innate immunity, NEDD8/RUB

Post-translational modification by ubiquitin plays a critical regulatory function in nearly all aspects of plant biology (Vierstra, 2009). Diverse conjugation enzymes attach monoubiquitin or polyubiquitin, with eight different linkages, as distinct signals to the regulatory and mechanistic components of various cellular processes. This ebook updates the functions, targets, and mechanisms of the conjugation components involved in the monoubiquitination of histones H2A and H2B and the polyubiquitination of all linkage types. Additionally, the roles and mechanisms of E3 ligases in biotic and abiotic stress responses and self-incompatibility (SI) and the regulation of cullin-based ligases (CRLs) by neddylation/deneddylation are updated. Finally, the functional roles of deubiquitination enzymes (DUBs) are reviewed together with a report on the biochemical and phylogenetic analyses of *Arabidopsis* OTU DUBs that support their functional differences.

The topology of the polyubiquitin chain produced by the ubiquitin E3 ligase determines the fate of the conjugated substrate. Here, Walsh and Sadanandom review the functional roles of different ubiquitin linkages in *Arabidopsis* (Walsh and Sadanandom, 2014). The conjugation components involved in the assembly of the K11-, K48-, and K63-linkages are conserved in plants. The K48-linked polyubiquitination targets regulatory factors for proteasomal degradation and is involved in diverse plant functions. The K63 linkage-forming RING E3s, RGLG1/2, are involved in auxin signaling, where they regulate auxin levels by affecting endocytic turnover of the auxin efflux transporter PIN2. In addition, plants overexpressing the K63-linkage-specific E2, UBC13, or harboring *rglg1*/*rglg2* double mutations exhibit bifurcated root hairs, showing an iron deficiency response. Two other reports also detail the importance of the K63 linkage in PIN2 turnover and iron deficiency response (Pan and Schmidt, 2014; Tomanov et al., 2014). While the Bachmair's group extends his discussion on K63-linkage in effector-triggered immunity, the second paper proposes that UBC13 and RGLGs are competed by DNA replication/repair under iron deficient conditions.

Histone H2A and H2B monoubiquitination represents distinct epigenetic marks that repress or activate transcription. Feng and Shen discuss the *Arabidopsis* E2s, E3s, and DUBs responsible for H2B monoubiquitination, which is crucial for the transcriptional activation of key regulators controlling flowering, seed dormancy, clock, photomorphogenesis, and pathogen defense (Feng and Shen, 2014). The mechanisms underlying the targeting of H2B monoubiquitination enzymes and transcriptional activation are updated. Conversely, H2A monoubiquitination, mediated by the polycomb repressive complex PRC1, is a repressive chromatin mark that is important for stem apical meristem maintenance, embryonic cell fate determinacy, and seed germination. The maintenance of gene repression also requires another polycomb complex PRC2, which is responsible for the Lys 27 methylation of histone H3 (H3K27me2/3). Interestingly, findings that challenge the paradigm of PRC2 and PRC1 sequential recruitment are discussed.

Duplan and Rivas update the functional roles of ubiquitin ligases in plant immune signaling (Duplan and Rivas, 2014). Ubiquitin E3 ligases are involved in pathogen perception, where they modulate pathogen-associated molecular pattern receptors at the plasma membrane or intracellular nucleotide-binding leucine-rich repeat-type receptors. These E3s are also involved in signaling responses downstream of pathogen perception through targeting and modulating vesicle trafficking components or transcription factors. Duplan and Rivas also discuss microbial effectors that target host E3s or act as E3s to counteract plant resistance. In parallel, Stone updates the functional roles of E3s in plant responses to abiotic stresses (Stone, 2014). Here E3s are involved in the suppression of stress response activators under non-stress conditions and the inactivation of response suppressors under stress. The roles of E3s in attenuating stress response signaling after stress relief are also discussed. Interestingly, this report outlines how multiple E3s and their targets are involved in the production and signaling of the stress-related hormone abscisic acid (ABA). Additionally, these reviews discuss the importance of the plant U-box armadillo repeat ligases (PUB-ARMs) in biotic and abiotic stress responses. Several PUB-ARMs target the plasma membrane or intracellular trafficking components. Moreover, Vogelmann et al. report that the *Arabidopsis* PUB-ARMs, SAUL1 (SENESCENCE-ASSOCIATED UBIQUITIN LIGASE 1) and its paralogs are plasma membrane (PM)-localized via their C-terminal ARM repeats (Vogelmann et al., 2014). PM-localization is conserved for SAUL1-type PUB-ARM orthologs in land plants, suggesting functional importance; however, their membrane targets have not yet been identified.

E3s are also involved in two major plant SI systems: *S* receptor kinase (SRK)-based and S-RNase-based. ARC1, a PUB-ARM E3, is critical for SRK-based SI in *Brassicaceae* plants. Indriolo and Goring provide updates on the conserved role of SRK-ARC1 signaling in self-pollen rejection in *Brassica* and *Arabidopsis* species and on the role of the ARC1 target, Exo71A1, in secretory activity during compatible pollination on the stigmatic papillae (Indriolo and Goring, 2014). ARC1 is proposed to negatively regulate Exo71A1 to disrupt secretion during incompatible pollination. *S* locus-encoded F-box proteins (SLFs), which are involved in S-RNase-based SI, are the substrate receptors for cullin1-based SCF E3s. Liu et al. provide evidence to support the cytoplasmic localization of pollen and pistil S-factors, SLFs and S-RNase in *Petunia hybrida* pollen tubes (Liu et al., 2014). They report a selective interaction between SLFs and S-RNase and non-self SLF-mediated S-RNase degradation in compatible but not incompatible pollen tubes. All evidence indicates that non-self SLFs mediate cytosolic S-RNase degradation to allow non-self-pollen acceptance. The self-SLF is unable to degrade S-RNase, which acts as a cytotoxin that rejects self-pollen.

CRLs represent the most prominent ubiquitin E3 ligase class, that is critical for specifying substrates, and are regulated by reversible NEDD8/RUB modification of the cullin subunit. Mergner and Schwechheimer update the critical components involved in NEDD8 processing, conjugation, and deconjugation in yeast, animals, and, in particular, plants (Mergner and Schwechheimer, 2014). The update provides insights on the role of cullin neddylation in auxin responses, the role of CSN-mediated cullin deneddylation and CSN-CRL interaction in photomorphogenesis and auxin responses, and the role of substrates, substrate receptors, CAND1, and CSN-associated proteins in CRL regulation. They also discuss possible non-cullin substrates and neddylation functions along with CRL regulation.

Five conserved classes of DUBs play important mechanistic roles in nearly all aspects of eukaryotic cellular processes. Isono and Nagel update the involvement of DUBs of all classes in various aspects of plant biology (Isono and Nagel, 2014). In most cases, the exact molecular mechanisms, targets, and cellular functions of plant DUBs require further investigation. The extent of conservation and divergence on mechanisms and cellular functions also require scrutiny for plant DUBs for which yeast and animal orthologs have been extensively described. In addition, Radjacommare et al. report the characterization of *Arabidopsis* OTU DUBs (Radjacommare et al., 2014), the corresponding mammal DUB class was recently identified as critical for various cellular processes and signaling pathways. Distinct biochemical properties and phylogenetic relationships support the involvement of *Arabidopsis* OTUs in conserved and also plant-specific cellular processes.

It is now clear that reversible ubiquitination is a critical regulatory element of numerous cellular processes and nearly all aspects of plant biology. Mechanistic studies for each ubiquitination or deubiquitination event, including target recognition, assembly and perception of diverse ubiquitin signals, and the regulation of the involved conjugation and deconjugation components could provide bases for better understanding of various aspects of plant biology. The small molecule-mediated substrate recognition of cullin-based CRLs identified in studies of auxin signaling is one excellent example (Santner et al., 2009). Timely updates are necessary in response to the rapid progress and important new discoveries obtained from functional and mechanistic studies of new substrates and the large numbers of conjugation and deconjugation components, such as ubiquitin E3 ligases and DUBs.

## Conflict of interest statement

The authors declare that the research was conducted in the absence of any commercial or financial relationships that could be construed as a potential conflict of interest.

## References

[B1] Duplan V. Rivas S. (2014). E3 ubiquitin-ligases and their target proteins during the regulation of plant innate immunity. Front. Plant Sci. 5:42. 10.3389/fpls.2014.00042 24592270 PMC3923142

[B2] Feng J. Shen W.-H. (2014). Dynamic regulation and function of histone monoubiquitination in plants. Front. Plant Sci. 5:83. 10.3389/fpls.2014.00083 24659991 PMC3952079

[B3] Indriolo E. Goring D. R. (2014). A conserved role for the ARC1 E3 ligase in Brassicaceae self-incompatibility. Front. Plant Sci. 5:181. 10.3389/fpls.2014.00181 24847339 PMC4017152

[B4] Isono E. Nagel M.-K. (2014). Deubiquitylating enzymes and their emerging role in plant biology. Front. Plant Sci. 5:56. 10.3389/fpls.2014.00056 24600466 PMC3928566

[B6] Liu W. Fan J. Li J. Song Y. Li Q. Zhang Y. E.. (2014). SCF^SLF^-mediated cytosolic degradation of S-RNase is required for cross-pollen compatibility in S-RNase-based self-incompatibility in *Petunia hybrida*. Front. Plant Sci. 5:228. 10.3389/fgene.2014.00228 25101113 PMC4106197

[B7] Mergner J. Schwechheimer C. (2014). The NEDD8 modification pathway in plants. Front. Plant Sci. 5:103. 10.3389/fpls.2014.00103 24711811 PMC3968751

[B8] Pan I.-C. Schmidt W. (2014). Functional implications of K63-linked ubiquitination in the iron deficiency response of *Arabidopsis* roots. Front. Plant Sci. 4:542. 10.3389/fpls.2013.00542 24427162 PMC3877749

[B9] Radjacommare R. Usharani R. Kuo C.-H. Fu H. (2014). Distinct phylogenetic relationships and biochemical properties of Arabidopsis ovarian tumor-related deubiquitinases support their functional differentiation. Front. Plant Sci. 5:84. 10.3389/fpls.2014.00084 24659992 PMC3950621

[B10] Santner A. Calderon-Villalobos L. I. A. Estelle M. (2009). Plant hormones are versatile chemical regulators of plant growth. Nat. Chem. Biol. 5, 301–307. 10.1038/nchembio.165 19377456

[B11] Stone S. L. (2014). The role of ubiquitin and the 26S proteasome in plant abiotic stress signaling. Front. Plant Sci. 5:135. 10.3389/fpls.2014.00135 24795732 PMC3997020

[B12] Tomanov K. Luschnig C. Bachmair A. (2014). Ubiquitin Lys 63 chains – second-most abundant, but poorly understood in plants. Front. Plant Sci. 5:15. 10.3389/fpls.2014.00015 24550925 PMC3907715

[B13] Vierstra R. D. (2009). The ubiquitin-26S proteasome system at the nexus of plant biology. Nat. Rev. Mol. Cell Biol. 10, 385–397. 10.1038/nrm2688 19424292

[B14] Vogelmann K. Subert C. Danzberger N. Drechsel G. Bergler J. Kotur T.. (2014). Plasma membrane-association of SAUL1-type plant U-box armadillo repeat proteins is conserved in land plants. Front. Plant Sci. 5:37. 10.3389/fpls.2014.00037 24600457 PMC3928556

[B15] Walsh C. K. Sadanandom A. (2014). Ubiquitin chain topology in plant cell signaling: a new facet to an evergreen story. Front. Plant Sci. 5:122. 10.3389/fpls.2014.00122 24744767 PMC3978257

